# Effect of nanohydrogel-containing *Ferula gummosa* resin on *Streptococcus mutans* and *Candida albicans*

**DOI:** 10.34172/joddd.025.41796

**Published:** 2025-06-30

**Authors:** Shima Golmohammadi, Behnaz Daraei, Ali Basir, Marzieh Rashidipour

**Affiliations:** ^1^Department of Periodontics, Faculty of Dentistry, Lorestan University of Medical Sciences, Khorramabad, Iran; ^2^Faculty of Dentistry, Lorestan University of Medical Sciences, Khorramabad, Iran; ^3^Department of Prosthodontics, Faculty of Dentistry, Lorestan University of Medical Sciences, Khorramabad, Iran; ^4^Razi Herbal Medicines Research Center, Lorestan University of Medical Sciences, Khorramabad, Iran

**Keywords:** *Candida albicans*, *Ferula*, Nanoparticles, Plant extracts, *Streptococcus mutans*

## Abstract

**Background.:**

Integrating nanoparticles with herbal medicine can enhance drug efficacy and leverage natural antimicrobial properties, addressing concerns related to drug side effects and microbial resistance. *Streptococcus mutans* and *Candida albicans*, key oral pathogens responsible for dental caries and candidiasis, pose significant clinical challenges. This study investigated the potential of *Ferula gummosa*-based nanoparticles in combating the oral pathogens *S. mutans* and *C. albicans*.

**Methods.:**

In this in vitro study, *F. gummosa* essential oil was extracted and analyzed using gas chromatography-mass spectrometry (GC-MS). A nanogel incorporating this oil was formulated with chitosan and tripolyphosphate (TPP). The physicochemical properties of the nanogel were characterized using scanning electron microscopy (SEM), Fourier transform infrared spectroscopy (FTIR), zeta potential analysis, dynamic light scattering (DLS), and atomic force microscopy (AFM). The minimum inhibitory concentration (MIC), minimum bactericidal concentration (MBC) against *S. mutans*, and minimum fungicidal concentration (MFC) against *C. albicans* were determined through microdilution assays.

**Results.:**

The major constituents of *F. gummosa* essential oil were identified as γ-cadinene (20.44%), T-cadinol (12.03%), sabinene (10.23%), and β-pinene (9.77%). The nanogel demonstrated efficient oil encapsulation, with an average nanoparticle size of 128.89±24.08 nm and a PDI of 0.226. The zeta potential of the nanoparticles increased upon oil incorporation. The MIC against *S. mutans* was 19.02 μg/mL for the nanogel and 781.25 μg/mL for the oil, while the MIC against *C. albicans* was 2.37 μg/mL for the nanogel and 195.31 μg/mL for the oil. MBC and MFC assays confirmed the enhanced antimicrobial efficacy of the nanogel.

**Conclusion.:**

*F. gummosa* essential oil exhibited significant antibacterial and antifungal properties. Formulating the oil into a nanostructure significantly enhanced its efficacy against *S. mutans* and *C. albicans*, presenting a promising alternative antimicrobial strategy.

## Introduction

 The growing interest in traditional “green medicine” is driven by concerns about the cost and side effects of synthetic drugs and the escalating resistance of pathogens to standard antibiotics.^1^ This has reignited enthusiasm for traditional medicinal plants, globally acknowledged for their therapeutic value.^2^ One such plant, *Ferula gummosa*, indigenous to Iran, is reputed for its anti-inflammatory and antioxidant effects.^3^ The proven efficacy of its root resin against certain bacteria and fungi further underscores its medicinal potential.^4,5^ Historically, *F. gummosa* has served various purposes, including traditional applications as an antiseptic, anti-seizure agent, anti-spasm remedy, pain reliever, inflammation reducer, and a tonic for memory enhancement. Recent investigations have substantiated its diverse therapeutic applications, including antimicrobial, anti-inflammatory, antinociceptive, antileptic, and spasmolytic properties.^6^

 Dental caries, a prevalent infectious oral disease, is closely associated with the formation of dental plaque biofilms.^7^ During the initial stages of dental caries development, the collaborative activity of enzymes (glucosyltransferase and fructosyltransferase) released by *Streptococcus mutans*, a key cariogenic bacteria, and adhesive-enhancing agents (glucans) secreted by *Candida albicans* creates environments conducive to the proliferation of other causative bacteria.^8^ *C. albicans*, in addition to its role in dental caries, is the microorganism responsible for oral candidiasis, the most prominent fungal disease of the oral cavity.^9^ Challenges, including resistance to antifungal treatments and side effects, have prompted researchers to explore new antifungal agents, especially those derived from natural compounds. Moreover, most oral diseases, including dental caries and periodontal diseases, result from biofilms whose structure shields pathogenic bacteria from antibiotics, posing a significant challenge to effectively controlling oral microorganisms. Traditional antimicrobial agents like chlorhexidine gluconate, used to minimize and prevent biofilm formation, come with drawbacks, including dark stains on teeth, alterations in oral microbiota, a burning sensation in the mouth, enhanced calculus formation, and changes in taste perception. Consequently, the intensified search for alternative solutions has led to herbal remedies and natural nanoparticles emerging as promising options for combating oral pathogens.

 The defensive barrier role of biofilm against conventional antimicrobial agents has led to the proposal of nanotechnology as an innovative strategy against microbe-related oral diseases. Nanoparticles’ unique physical and chemical characteristics, such as significantly small size and increased surface-to-mass ratio, enhance their antimicrobial properties.^10^ Nanomedicine is an innovative approach to enhance the efficiency of herbal constructs.^11^ The use of natural biopolymers like chitosan as drug carriers in nanoparticles proves effective in harnessing the medicinal properties of plants, offering advantages such as biodegradability, biocompatibility, low toxicity, and enhanced safety.^12^ Additionally, the incorporation of natural antimicrobial agents into chitosan amplifies its antimicrobial properties, substantially improving the targeted delivery of therapeutic molecules to cells and tissues. This approach enables a slow and sustained release, optimizing overall effectiveness.^13^

 Due to the multi-faceted therapeutic properties of *F. gummosa*, it has attracted attention as a promising candidate for controlling oral pathogens.^14^ While studies have investigated the effects of *F. gummosa* resin on various microorganisms,^5,15^ none have applied nanotechnology to improve the bioavailability and efficacy of the essential oil derived from various parts of the plant. This study explored the potential of nanohydrogel-containing *F. gummosa* resin as a natural therapeutic agent against two major dental pathogens, *S. mutans* and *C. albicans*.

## Methods

###  Plant preparation and oleo-resin extraction

*Ferula gummosa* was initially obtained from a local herbal market. It was then identified by a herbarium center at a university, and the voucher herbarium code PMP-1859 was assigned. The resin was collected by making incisions on the rhizome or upper roots of the plant and stored in a dry, shaded environment away from direct sunlight. Additionally, to increase the contact surface and reduce its volume, the obtained resin was dried and finely ground using a grinder.

###  Essential oil 

*Ferula gummosa* essential oil was obtained using the distillation method. In this process, 100 grams of *F. gummosa* resin were immersed in one liter of distilled water and subjected to hydro-distillation within the Clevenger apparatus for 3 hours. Subsequently, the resulting essential oil was stored at 4 °C until the following experimental stages.

###  Gas chromatography-mass spectrometry analysis (GC-MS)

 GC-MS analysis was conducted using an AGILENT 7890A gas chromatograph combined with a mass spectrometer (Model 5975C, Agilent Technologies, Santa Clara, CA, USA) to analyze volatile and semi-volatile compounds within the essential oil. A GC capillary column, SGE-BP20 (composed of phenyl methyl siloxane with a length of 30 meters, an internal diameter of 250 μm, and a film thickness of 0.25 μm, Agilent Technologies), was employed. The oven temperature was programmed to increase from 60 ºC to 230 ºCat a rate of 8 ºC/min. The injection port temperature was held at 280 ºC, and helium gas served as the carrier gas with a flow rate of 1.3 mL/min. Mass spectrometry was performed using electron ionization at an energy level of 70 eV within a mass range of 20‒550 Da. The constituents of the *F. gummosa* essential oil were identified by comparing their Kováts retention indices (KI) and mass spectra with known compounds available in the NIST 2014 library.^16^

###  Nanohydrogel preparation

 The research aimed to encapsulate *F. gummosa* resin oil within chitosan nanoparticles (CSNPs) and used tripolyphosphate (TPP) as a cross-linking agent to fabricate *F. gummosa* resin oil-loaded CSNPs. Separate solutions of 0.5% chitosan and 0.3% TPP were meticulously prepared for the experiment. Initially, 0.1 grams of low molecular weight chitosan was gradually introduced into 20 mL of a 1% acetic acid solution while continuously stirring for 2 hours. Subsequently, 5 mL of Tween 20 emulsifier was incorporated, and the mixture was stirred for an additional 2 hours. Following this, 1 mL of *F. gummosa* resin oil was introduced slowly and drop by drop over 20 minutes into the chitosan acidic solution while ensuring continuous stirring. Finally, 5 mL of an 0.3% TPP solution was added to induce the formation of a hydrogel network. This particular hydrogel, characterized by its nano-sized cavities, was achieved through ionotropic gelation. High-speed centrifugation was then executed to separate the supernatant solution containing the nanohydrogel from the sedimented phase.

 In a parallel experiment, a blank nanohydrogel, i.e., without including *F. gummosa* resin oil, was synthesized using identical procedures and proportions as outlined above.

###  Tests performed on nanohydrogel

####  UV-vis spectrophotometry

 The encapsulation efficiency of *F. gummosa* in the nanohydrogel was assessed using UV-Vis spectroscopy with a UV-Vis spectrophotometer (UV-1900, Shimadzu, Kyoto, Japan). This instrument uses lamps emitting light in the visible (tungsten lamp) and UV (deuterium lamp) regions, covering wavelengths from 190 nm to 900 nm. It enables the analysis of samples based on their light-absorbing properties. The maximum wavelength of light absorption (λmax) for *F. gummosa* resin oil was determined, and subsequently, the optical density (OD) at this specific wavelength was measured for various concentrations of *F. gummosa* resin oil and nanohydrogel containing the resin oil. These OD values were used to construct a calibration curve, facilitating precise quantification of *F. gummosa* resin oil concentration within the nanohydrogel.

 Encapsulation efficiency = (mass of loaded *F. gummosa*/mass of initial *F. gummosa*) × 100

####  Scanning electron microscopy (SEM)

 The morphology of the synthesized nanoparticles was studied using SEM. Freeze-dried nanoparticles were positioned on a gold-coated stub and examined at 15 kV with a 6300-field emission scanning electron microscope (Hitachi, S-4160).

####  Zeta potential and dynamic light scattering (DLS) analysis

 Zeta potential analysis was employed to determine the electric charge of the particles using a Malvern Zetasizer Nano-range instrument (Malvern Instruments Ltd., Malvern, UK). Additionally, DLS analysis was performed to measure particle sizes, which involved observing laser light interactions with the particles, recording scattering patterns, and capturing variations in light intensity due to the Brownian motion of the particles.

####  Fourier transform infrared spectroscopy (FTIR)

 FTIR spectroscopy was conducted to identify functional groups and elucidate the structure of the organic compounds. IR light was directed at the three test materials: *F. gummosa* resin essential oil, nanohydrogel containing *F. gummosa* resin oil, and the blank nanohydrogel (comprising chitosan and TPP). Functional groups were identified based on the absorption or transmission of IR radiation using the BRUKER TENSOR 27 instrument (Germany).

####  Atomic force microscopy (AFM)

 AFM was used to analyze the surface topography of the samples. Using electrostatic interactions between the atoms at the tip and those on the sample surface, the tip exhibited upward and downward deflections. These deflections were detected by a laser and photodiode integrated into the microscope, facilitating the creation of three-dimensional images of the samples at nanoscale dimensions. AFM analysis was performed on 200 microliters of nanohydrogels containing *F. gummosa* resin oil and blank nanohydrogels, deposited on a glass slide, and examined after air-drying at room temperature, using the AFM instrument model of 0101/A (Ara Research Co Nano Experts, Iran).

###  Microbial and fungal specimens

 The selected standard strains of *S. mutans* (ATCC35668) and *C. albicans* (ATCC10231) were purchased from the National Center for Genetic and Biological Resources of Iran.

###  Minimum inhibitory concentration (MIC) and minimum bactericidal concentration (MBC) 

 The MIC and MBC for *S. mutans* and the MIC and MFC for *C. albicans* were determined using the microdilution method in sterile 96-well plates, following the protocol established by the Clinical and Laboratory Standards Institute (CLSI).^17^

 Initially, stock solutions for the three study groups were prepared, including *F. gummosa* essential oil, nanohydrogel containing *F. gummosa* essential oil, and blank nanohydrogel. A sterile culture medium was also prepared, employing brain-heart infusion (BHI) for *S. mutans* and Sabouraud dextrose broth for *C. albicans*. The stock solution of *F. gummosa* essential oil was set at a concentration of 200 000 µg/mL. In comparison, the stock solutions of the nanohydrogel were prepared at a concentration of 120 000 µg/mL, based on the total weight of the nanohydrogels.

 Subsequently, 100 µL of the sterile culture medium was added to rows three through twelve of the 96-well plates. In the first two rows, 100 µL was introduced from the prepared bacterial inoculum, initiating a serial dilution process from the second to the tenth row. This dilution involved transferring 100 µL from the second row to the third row and 50 µL from the third row to the fourth through the tenth row. In rows two to ten, 10 µL of a 24-hour microbial culture, equivalent to 0.5 McFarland turbidity (1.5 × 10^8^ CFU/mL), was added.

 The antimicrobial effect of *F. gummosa* essential oil was evaluated in the 96-well plates at concentrations of 100,000 µg/mL for the essential oil and 60 000 µg/mL for the nanohydrogels. These plates were then incubated for 24 hours at 37 °C.

 Following incubation, 2,3,5-triphenyl tetrazolium chloride (TTC) was used as a visual indicator for bacterial growth. Wells that exhibited no change in color were designated as the MIC. Colorless wells were subjected to sub-culturing on Mueller-Hinton agar medium for *S. mutans* and Sabouraud dextrose agar for *C. albicans* to determine the MBC and MFC, respectively. The negative control consisted of dimethyl sulfoxide (DMSO), while 0.2% chlorhexidine (CHX) was used as the positive control for *S. mutans*, and 100 000-IU/mL nystatin served as the positive control for *C. albicans*.

###  Data analysis

 All measurements were conducted three times. One-way analysis of variance (ANOVA) in SPSS 23 (SPSS Inc., Chicago, IL, USA) was used to determine the statistical significance of the experimental results. In the cell viability test, significance was attributed to *P *values < 0.05.

## Results

Table 1 summarizes the essential oil constituents and their respective percentages. The viscosity, dielectric constant, and zeta potential of the *F. gummosa* nanohydrogel were compared to those of the blank nanohydrogel, as summarized in Table 2.

**Table 1:** Chemical components (%) of *F. gummosa* essential oil was analyzed via GC-MS

**No.**	**Compound**	**Retention time (min)**	**Content (%)**
1	α-Pinene	1.43	3.23
2	β-Pinene	1.51	9.77
3	Sabinene	1.6	10.23
4	o-Cymene	2.86	1.94
5	α-Copaene	5.72	5.7
6	Germacren D	6.44	0.63
7	Pinocarvone	6.78	1.39
8	Aromandendrene	7.31	2.39
9	Terpinen-4-ol	7.56	7.56
10	cis-Muurola-4(15),5- diene	8.8	8.8
11	Epizonarene	9.83	1.07
12	α-Muurolene	10.161	1.48
13	γ-Cadinene	10.95	20.44
14	Unknown	16.42	5.9
15	Epicubenol	18.39	1.34
16	Guaiol	19.06	1.53
17	T-Cadinol	20.22	12.03
18	Bulnesol	20.78	1.86
19	α-Cadinol	21.03	0.62
Total identified			92.91

**Table 2:** Viscosity, dielectric constant, and zeta mean comparison between blank nanohydrogel and *F. gummosa* nanohydrogel

**Parameter**	**Blank nanohydrogel**	***F. *** ***gummosa*** ** nanohydrogel**
Viscosity	0.9307 MPa	0.9472 MPa
Dielectric constant	79.10	79.37
Zeta mean	0.04 ± 1.70 mV	-7.38 ± 9.57 mV

**Figure 1:**
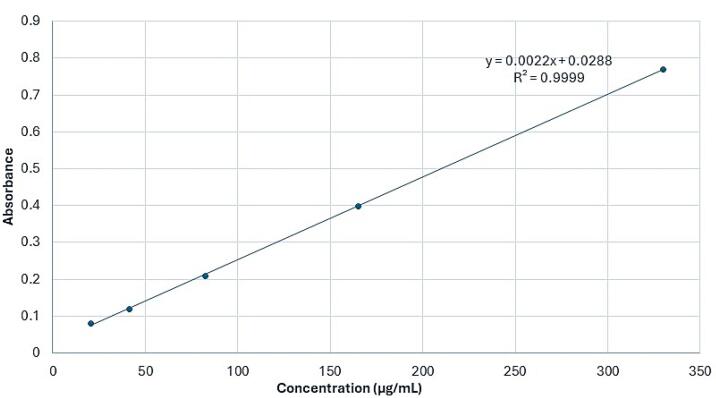
Calibration curve of *F. gummosa* essential oil absorbance at varying concentrations (λₐₓ = 259 nm), using UV-Vis spectrophotometry to quantify essential oil in the nanohydrogel formulation

 The maximum light absorption (λmax) of the *F. gummosa* essential oil was observed at a wavelength of 259 nm. Using UV-Vis spectrophotometry, a calibration curve was generated from OD measurements at five different essential oil concentrations. After determining the OD of a specific concentration of the nanohydrogel containing *F. gummosa* resin essential oil, the concentration of resin essential oil within the nanohydrogel was quantified using the derived formula (y = 0.0022x + 0.0288). The OD of *F. gummosa* resin essential oil at a concentration of 50.000 µg/mL yielded a measurement of 0.168. Substituting this value into the formula (in place of ‘y’) allowed calculating the resin essential oil concentration in the nanohydrogel, resulting in 43.63 µg/mL. These calculations revealed that the concentration of *F. gummosa* essential oil in the stock solution, prepared for the evaluation of its antimicrobial properties, was determined to be 2.152 µg/mL (equivalent to 0.6 grams of the nanohydrogel containing *F. gummosa* essential oil dissolved in 5 mL of distilled water). Figure 1 illustrates the calibration curve based on the absorbance of *F. gummosa* essential oil at varying concentrations.

**Figure 2:**
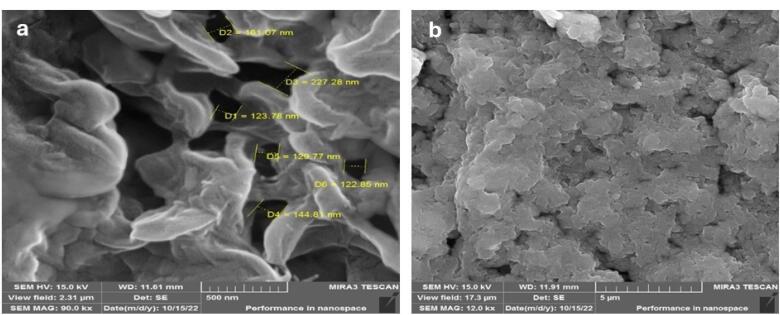
SEM images of (a) blank nanohydrogel at 90 000 × and (b) nanohydrogel loaded with *F. gummosa* essential oil at 12 000 × magnification. The loaded formulation shows a denser, more compact surface, indicating successful oil encapsulation (scale bars: 500 nm (a), 5 µm (b))

**Figure 3:**
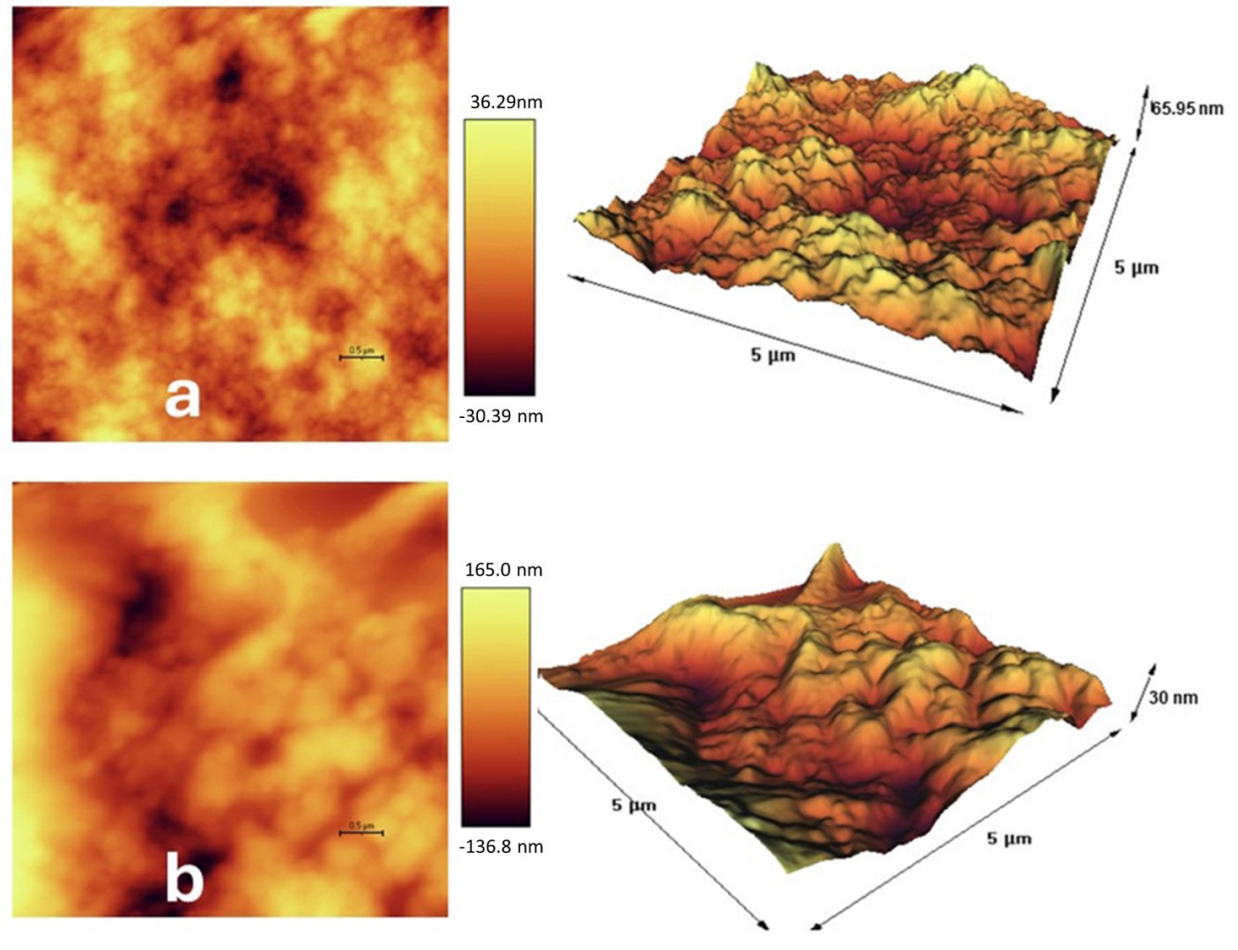
AFM surface topography of (a) blank nanohydrogel and (b) nanohydrogel loaded with *F. gummosa* essential oil. Increased surface roughness in (b) may contribute to enhanced antimicrobial interaction with microbial membranes. (scale bars = 0.5 µm (2D images) and 5 µm (3D images))

 SEM and AFM views of the blank nanohydrogel and the nanohydrogel loaded with *F. gummosa* resin oil are depicted in Figures 2 and 3.

**Figure 4:**
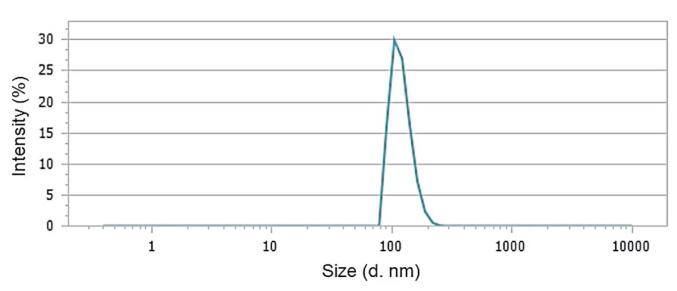
Dynamic light scattering (DLS) analysis of *F. gummosa* nanohydrogel showing an average particle size of 128.89 ± 24.8 nm and a PDI of 0.226, indicating monodisperse nanoparticle distribution suitable for improved bioavailability and interaction with microbial cells

 The DLS data, as depicted in Figure 4, indicated that the average particle size (Z-ave) within the *F. gummosa* resin oil-containing nanohydrogel was measured at 128.89 ± 24.8. Furthermore, the polydispersity index (PDI) for the nanohydrogel containing *F. gummosa* resin oil was reported as 0.226, while the blank nanohydrogel exhibited a PDI of 0.792.

**Figure 5:**
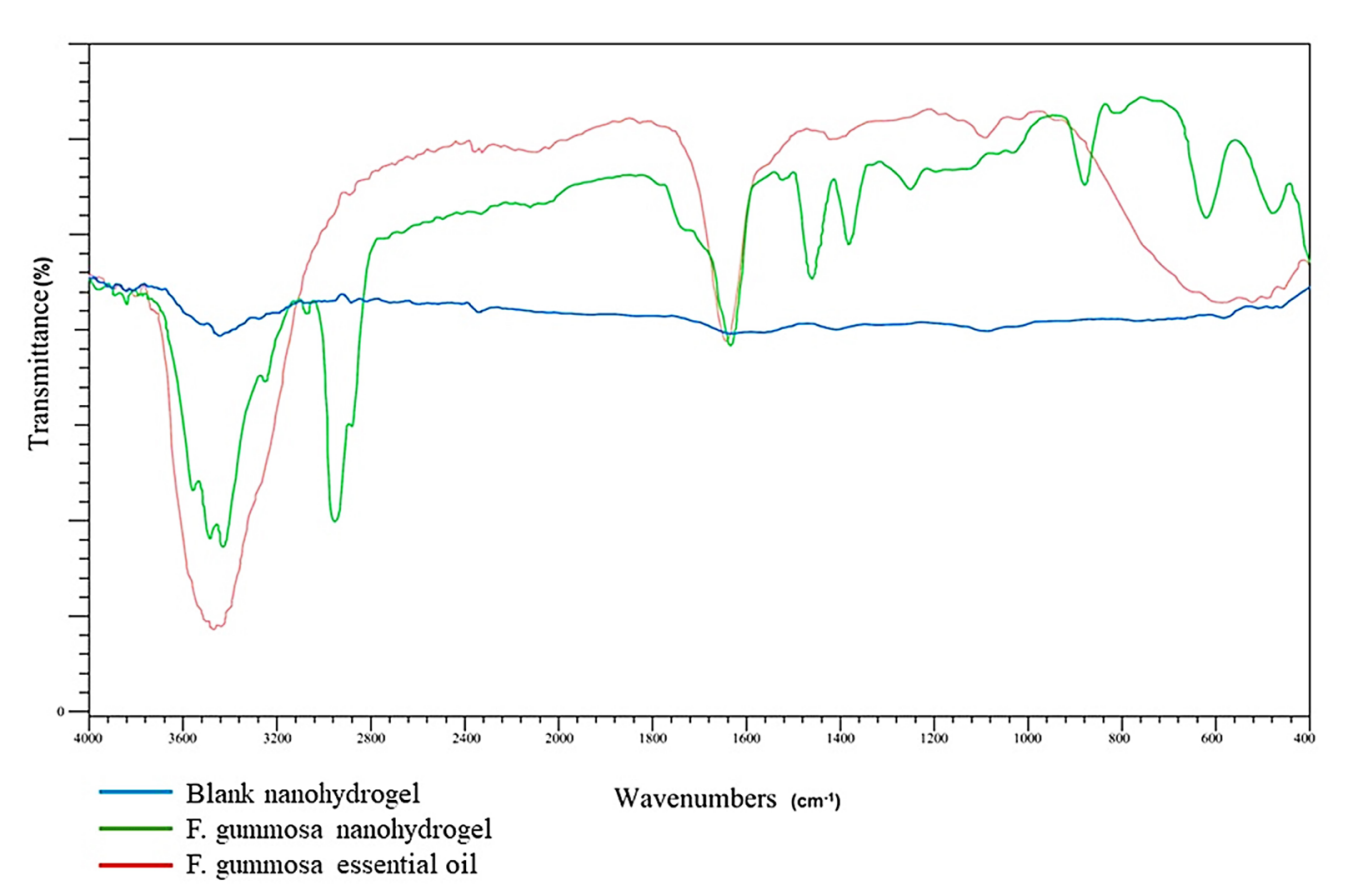
FTIR spectra comparing blank nanohydrogel, *F. gummosa* nanohydrogel, and *F. gummosa* essential oil. Shifts and overlaps in characteristic peaks (e.g., –OH, C = O, C–O–C) indicate the successful incorporation of the oil into the nanohydrogel matrix and suggest interactions involved in encapsulation at the molecular level

Figure 5 exhibits the FTIR spectra of blank nanohydrogel, the nanohydrogel loaded with *F. gummosa* and *F. gummosa* essential oil, emphasizing their distinct infrared transmittance spectral patterns.

Tables 3 and 4 present the MIC and MBC of *F. gummosa* resin essential oil, nanohydrogels, and positive and negative control substances against *S. mutans*, respectively.

**Table 3:** MIC and MBC against *Streptococcus mutans* for study groups and controls

**Group**	**MIC (µg/mL)**	**95% CI (MIC)**	***P*** **-value (MIC)**	**MBC (µg/mL)**	**95% CI (MBC)**	***P*** ** value (MBC)**
Blank nanohydrogel	30000	28500–31500	0.0013	60000	57000–63000	0.0011
*F. gummosa* nanohydrogel	19.02	18.1–20.0		19.02	18.4–19.6	
*F. gummosa* essential oil	781.25	740–823		781.25	750–812	
CHX	0.98	0.93–1.03		1.96	1.85–2.07	
DMSO	Growth in all concentrations	-		-	-	

**Table 4:** MIC and MFC against *Candida albicans* for study groups and controls

	**MIC (µg/mL)**	**95% CI (MIC)**	***P*** ** value (MIC)**	**MFC (µg/mL)**	**95% CI (MFC)**	***P*** ** value (MFC)**
Blank nanohydrogel	3750	3575–3925	0.0027	7500	7150–7850	0.0021
*F. gummosa* nanohydrogel	2.37	2.25–2.50		4.75	4.50–5.00	
*F. gummosa* essential oil	195.31	185–206		195.31	185–206	
Nystatin	1.22	1.15–1.29		1.22	1.15–1.29	
DMSO	Growth in all concentrations	-		-	-	

 DMSO and nystatin served as negative and positive controls, respectively. Subsequently, the contents of the wells without growth in the MIC test were cultured on a solid Mueller-Hinton agar medium. After 24 hours of anaerobic incubation, the MBC results were examined. The MIC of nystatin against *C. albicans*, determined by microdilution, was 22.1 µg/mL. Considering that the concentration of nystatin is 100 000 IU/mL, and according to the International Units converter, each IU of nystatin is equivalent to 0.2 µg, the nystatin concentration is 20 000 µg/mL. Since this concentration was halved with an equal volume of the culture medium, the examination of nystatin concentrations began at 10 000 µg/mL.

## Discussion

 Our investigation into the essential oil of *F. gummosa* resin, using GC-MS, revealed a robust composition with notable components, including γ-cadinene, T-cadinol, sabinene, and β-pinene. These constituents, recognized for their antimicrobial properties, have been extensively studied for their efficacy against common oral pathogens, including *S. mutans* and *C. albicans*.^18-23^ This study focused on harnessing these properties by developing a nanohydrogel incorporating *F. gummosa* resin essential oil, with a specific emphasis on its potential impact on antimicrobial properties.

 The microscopic analysis illustrated nanoscale-sized voids in the blank nanohydrogel formed due to electrostatic interactions. These voids were filled after adding *F. gummosa* resin essential oil, resulting in a compact structure.

 The zeta potential of the nanohydrogel containing *F. gummosa* resin essential oil was -7.38 mV, indicating improved particle dispersion and stability compared to the blank nanohydrogel (-0.04 mV). DLS revealed an average particle size (Z-ave) of approximately 128.89 ± 24.8 nm for the nanohydrogel containing *F. gummosa* resin essential oil. The PDI was measured at 0.226, indicating reduced particle aggregation. In contrast, the blank nanohydrogel had a PDI of 0.792, suggesting increased particle aggregation.

 Further characterization through FTIR spectra confirmed the successful encapsulation of *F. gummosa* resin essential oil within the nanohydrogel, with specific functional groups corresponding to essential oil peaks not visible.

 Three-dimensional AFM images showed a textured surface with varying elevations in the nanohydrogel containing *F. gummosa* resin essential oil. This surface roughness may enhance adhesion to bacteria associated with dental decay, such as *S. mutans*.

 Our study unequivocally demonstrated the potent bacteriostatic and bactericidal properties of *F. gummosa* resin essential oil against *S. mutans*. Additionally, the essential oil displayed notable antifungal properties against *C. albicans*. The integration of the essential oil into a nanogel formulation significantly enhanced its antimicrobial effectiveness, as evidenced by reduced MIC and MBC values compared to the blank nanogel. Comparing our findings with Abbaszadegan et al,^5^ we observed consistent antibacterial activity against various oral pathogens, including *C. albicans*, with an MIC against *C. albicans* reported as 50 µg/mL. Variations in antimicrobial properties between studies underscored the diversity of active compounds in plants from different regions. Furthermore, compared to other studies exploring chitosan and chitosan-based nanoparticles, our study’s effectiveness against *S. mutans* and *C. albicans* suggested a promising alternative with lower concentrations required for efficacy.

 Recent findings in nanotechnology-based phytochemical delivery further support our results. For instance, albumin nanoparticles loaded with *Mentha* extract exhibited enhanced antibacterial activity against methicillin-resistant *Staphylococcus aureus* (MRSA) compared to the free extract while maintaining lower cytotoxicity.^24^ Similarly, silver nanoparticles biosynthesized using *Musa paradisiaca* (banana) and *Citrus sinensis* (orange) peel extracts showed strong antimicrobial effects against multidrug-resistant strains such as *Acinetobacter baumannii* and *Klebsiella pneumoniae.*^25^ These examples underscore the role of nanocarrier systems in improving the performance of natural compounds by enhancing their bioavailability, stability, and cellular delivery. Consistent with these findings, our study demonstrated that incorporating *F. gummosa* essential oil into a nanohydrogel formulation significantly enhanced its antimicrobial efficacy against *S. mutans* and *C. albicans*. Unlike studies comparing different plant species, we focused on various formulations of *F. gummosa* to control for interspecies variability and isolate the effect of nanoencapsulation.

 The findings of the present study unequivocally demonstrated the bacteriostatic and bactericidal properties of *F. gummosa* resin essential oil against *S. mutans*. Furthermore, the *F. gummosa* resin essential oil exhibited notable antifungal properties against *C. albicans*. Importantly, integrating *F. gummosa* resin essential oil into a nanogel formulation significantly amplified its antimicrobial effectiveness against these oral pathogens. This enhancement is evident in the reduced MIC and MBC values, measuring 19.02 µg/mL for *S. mutans* and 2.37 µg/mL and 4.75 µg/mL for *C. albicans*, respectively. While chitosan itself demonstrates efficacy against oral microorganisms,^26,27^ it is essential to highlight that the blank nanogel displayed inhibitory and bactericidal effects, albeit at significantly higher concentrations against these pathogens.

 Various studies have highlighted the diverse applications of *F. gummosa*, including its antibacterial properties, incorporation into liposomes, and the synthesis of nanocrystals with potential applications in bioimaging. Our research stands out by focusing on loading *F. gummosa* resin essential oil onto nanoparticles and investigating their specific effects on *S. mutans* and *C. albicans*.

 Sepahi et al^28^ explored the antibacterial and non-hemolytic properties of aqueous extracts derived from *F. gummosa* against *Staphylococcus aureus* and *Escherichia coli*. They reported significant antibacterial effects, with MIC values below 750 μg/mL and no notable hemolytic activity. Notably, unlike our study, Sepahi et al did not specify the plant part used. Nazemisalman et al^29^ reported that *F. gummosa* essential oil possesses antibacterial efficacy against *Enterococcus faecalis*, comparable to CHX, indicating its potential for clinical use in addressing oral and dental pathogens.

 Several studies have explored diverse applications of *F. gummosa*, showcasing its versatility and potential in various fields. Najafi et al.^30^ loaded liposomes with *F. gummosa* essential oil, emphasizing heightened antibacterial activity against *E. coli*, particularly at subinhibitory concentrations. This work aligns with the investigation by Kamelnia et al,^31^ who produced cellulose nanocrystals from *F. gummosa*, demonstrating no cytotoxic effects on A549 cells and stability in radiolabeling (Tc-99m), underscoring the potential of *F. gummosa*-derived materials in bioimaging applications.

 In a cytotoxicity context, Mousavi-Kouhi et al^32^ synthesized gold-coated nanoceria (Au/nanoceria) using *F. gummosa* gum as a capping agent. The study highlighted a notable toxicological impact on breast cancer cell lines (MCF7) with minimal effects on normal cells, emphasizing the dose and time-dependent nature of nanoceria toxicity. Similarly, Hosseini et al^33^ reported cytotoxic and apoptotic effects of *F. gummosa* gum extract on renal cell carcinoma cell line (ACHN). Forouzmand et al^34^ also demonstrated that *F. gummosa* enhanced cytotoxicity in HeLa cells through apoptosis induction and, when co-administered with radiotherapy, significantly increased radiosensitivity, suggesting its potential as a valuable radiosensitizer agent for cervical cancer treatment.

 For diabetic control, *F. gummosa* essential oil-loaded nano-fibers exhibit inhibitory effects on α-amylase and α-glucosidase, maintaining the herb’s antioxidant activity.^35^ Additionally, administering the ethanolic extract of *F. gummosa* oleo-resin to diabetic rats^36^ yields antihyperglycemic effects, including reduced fasting blood glucose levels, alleviation of oxidative stress-induced damage in the liver and kidneys, and restoration of antioxidant enzyme activity.

 While our findings are promising, additional research is recommended to evaluate the efficacy of *F. gummosa* resin essential oil and its nanogels against a broader spectrum of oral pathogens. Furthermore, assessing cellular toxicity associated with the essential oil and corresponding nanogels is crucial for a comprehensive understanding. Future studies should explore the effectiveness of essential oil and its nanogel formulation through animal studies or clinical trials.

## Conclusion

*Ferula gummosa* resin essential oil demonstrated promising antimicrobial characteristics, encompassing both bacteriostatic and bactericidal effects. Due to these valuable attributes, it emerges as a potential natural remedy for infectious oral diseases linked to *S. mutans*, including dental caries and those associated with *C. albicans*, specifically various forms of oral candidiasis. The transformation of this essential oil into nanogel form holds the promise of substantially augmenting its antimicrobial efficacy against the diseases mentioned above.

## Competing Interests

 None declared.

## Ethical Approval

 “Consent to Participate” was not applicable as it is an in vitro study conducted in a laboratory setting. The ethics code is IR.LUMS.REC.1401.071.
